# Nonclinical and clinical characterization of the absorption, metabolism, and excretion of islatravir

**DOI:** 10.1128/aac.01030-24

**Published:** 2025-01-14

**Authors:** Kerry L. Fillgrove, Randolph P. Matthews, Bing Lu, Yuexia Liang, Munjal Patel, Wen Liu, Catherine Z. Matthews, Yang Liu, S. Aubrey Stoch, Rosa I. Sanchez, Marian Iwamoto

**Affiliations:** 1 Merck & Co., Inc 2793 , Rahway, New Jersey, USA; Providence Portland Medical Center, Portland, Oregon, USA

**Keywords:** nucleoside reverse transcriptase translocation inhibition, human immunodeficiency virus, absorption, metabolism, excretion, phase 1, pharmacokinetics, mass balance, metabolite profiling

## Abstract

The development of new and improved antiretroviral therapies that allow for alternative dosing schedules is needed for people living with HIV-1. Islatravir is a deoxyadenosine analog in development for the treatment of HIV-1 that suppresses HIV-1 replication via multiple mechanisms of action, including reverse transcriptase translocation inhibition and delayed chain termination. Islatravir is differentiated from other HIV-1 antiretrovirals by its high potency, long *t*_½_, broad tissue distribution, and favorable drug resistance profile. A comprehensive evaluation was performed to provide data on the mass balance, absorption, metabolism, and excretion of islatravir through studies in nonclinical species, and in adults without HIV-1 infection, using radiolabeled islatravir. Islatravir was well absorbed in both nonclinical species and humans following oral administration. The elimination of islatravir occurs primarily by a combination of oxidative deamination to 4′-ethynyl-2-fluoro-2′-deoxyinosine and renal excretion of unchanged islatravir. Islatravir and 4′-ethynyl-2-fluoro-2′-deoxyinosine are the major circulating drug components in all species assessed. Islatravir is readily taken up into cells with efficient phosphorylation to the mono-, di-, and triphosphate forms. The pharmacologically active islatravir triphosphate is the most abundant intracellular phosphorylated species, as shown by the results of *ex vivo* studies. This characterization of the absorption, metabolism, and elimination of islatravir in humans and nonclinical species supports its further development for the treatment of HIV-1.

## INTRODUCTION

Antiretroviral therapy plays an important role in reducing the mortality and morbidity associated with HIV-1 infection, as well as reducing transmission rates (1). Several agents and fixed-dose combination regimens are available for the treatment of HIV infection; however, limitations to available therapies include safety and tolerability issues and the emergence of drug-resistant variants (2). Development of new and improved antiretroviral agents for HIV-1 infection, including those that allow for alternative dosing schedules, is therefore warranted.

**Fig 1:**
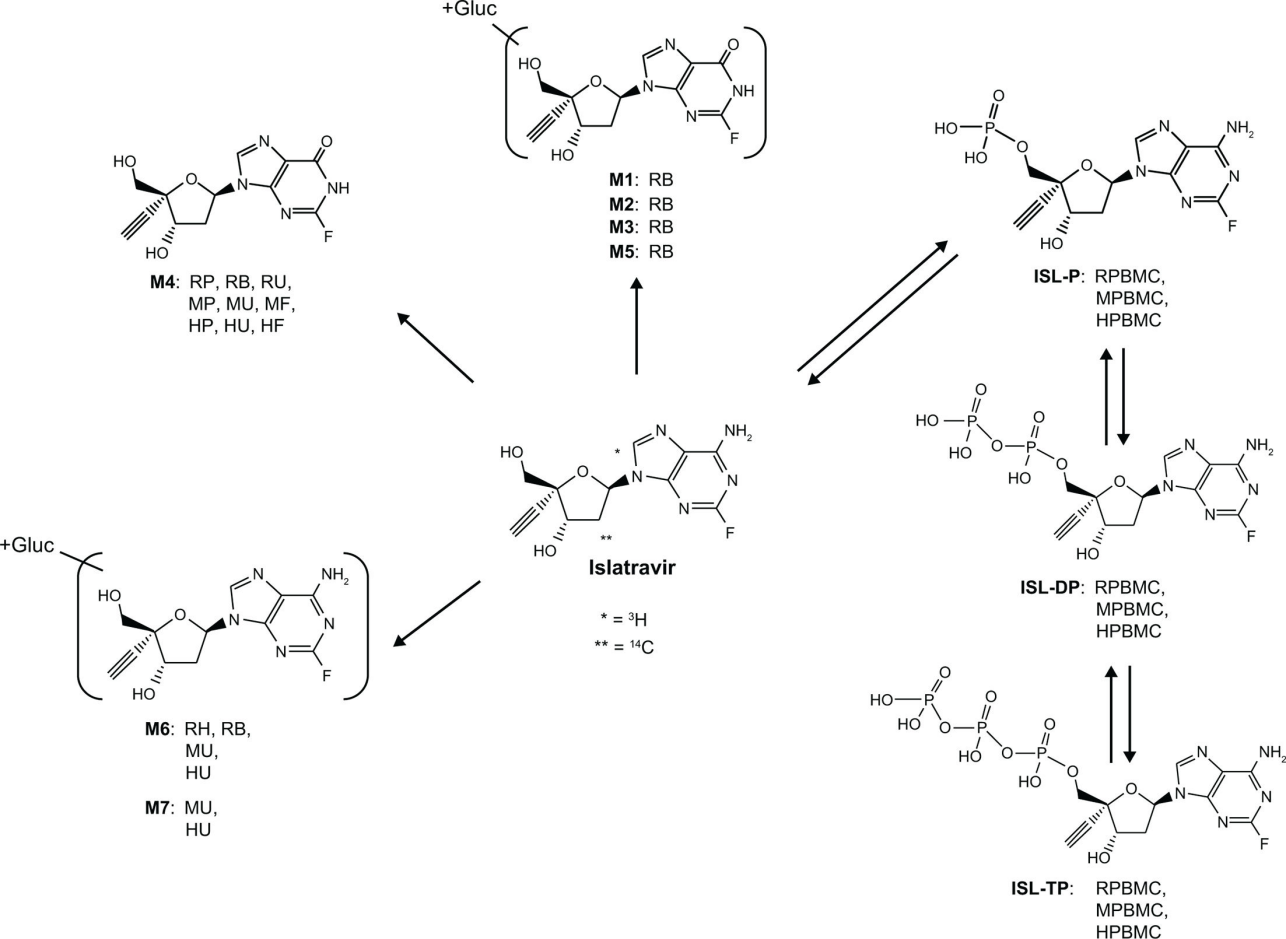
Proposed metabolites of ISL in *in vivo* and *in vitro* systems. + Gluc, glucuronide conjunction; HF, human feces; HPBMC, human peripheral blood mononuclear cell; HU, human urine; ISL, islatravir; ISL-DP, ISL diphosphate; ISL-P, ISL monophosphate; ISL-TP, ISL triphosphate; MF, monkey feces; MP, monkey plasma; MPBMC, monkey peripheral blood mononuclear cell; MU, monkey urine; RB, rat bile; RP, rat plasma; RPBMC, rat peripheral blood mononuclear cell; RU, rat urine.

Islatravir (ISL, MK-8591) is a deoxyadenosine analog under investigation for the treatment of HIV-1 in daily and weekly dosing regimens (3–6). The structure of ISL (Fig. 1) leads to inhibition of HIV-1 replication by multiple mechanisms of action, including reverse transcriptase translocation inhibition and delayed chain termination, which contribute to the high potency of ISL against HIV-1 (and drug-resistant variants) and its high barrier to resistance (5, 7–9).

The efficacy of ISL has been assessed in treatment-naive adults with HIV as monotherapy (10) and in combination with doravirine, demonstrating effective viral suppression (6). Following oral administration in humans, ISL is rapidly absorbed; following cellular uptake, ISL is phosphorylated to its active form, islatravir-triphosphate (ISL-TP), which interferes with HIV reverse transcriptase activity (11).

ISL is eliminated as unchanged drug by renal excretion (12, 13), with nonclinical studies demonstrating that approximately 30%–60% of the ISL total plasma clearance is attributed to renal excretion (12). Results from *in vitro* studies suggest that ISL is not a substrate of renal transporters, and therefore, renal excretion of ISL is likely mediated primarily via glomerular filtration (12, 14). Nonclinical data demonstrate that 4′-ethynyl-2-fluoro-2′-deoxyinosine (M4) is the major metabolite of ISL (12), and M4 is also excreted in the urine in humans (12, 15).

Understanding the metabolism and disposition of new therapeutic agents is an integral part of the pharmacokinetic (PK) and safety evaluation of a drug. A comprehensive evaluation of ISL was performed to provide data on the absorption, metabolism, and excretion of ISL through mass balance and metabolite profiling studies in nonclinical species and adults without HIV-1 infection, using radiolabeled ISL.

## RESULTS

### Excretion of radioactivity in nonclinical species

Following an oral dose of [^3^H]ISL in rats, the average total radioactivity recovered in the excreta, within 72 hours, represented 91.1% of the administered dose (Table 1). Most of the radioactivity was recovered in urine (73.7%), with smaller amounts being recovered in bile and feces (8.3% and 4.7%, respectively). In rhesus monkeys, the average total radioactivity recovered in the excreta within 120 hours represented 82.4% of the administered dose. Much of the radioactivity was recovered in urine (46.6%), followed by feces (31.0%), with small amounts recovered in bile (3.6%; Table 1).

**TABLE 1:** Mean percentage of radioactivity recovered following single 5-mg/kg oral-dose administration of [^3^H]ISL in rats and monkeys

Animal model	Fraction of radioactivity recovery, mean % (SD)				
	Urine	Feces	Bile	Cage wash/wipes	Total
Rats (*n* = 3)	73.7 (15.9)	4.7 (1.0)	8.3 (3.3)	4.4 (4.4)	91.1 (10.0)
Rhesus monkeys (*n* = 3)	46.6 (8.7)	31.0 (8.3)	3.6 (1.7)	1.2 (0.3)	82.4 (2.4)

### Metabolite profiles in nonclinical species

**Fig 2:**
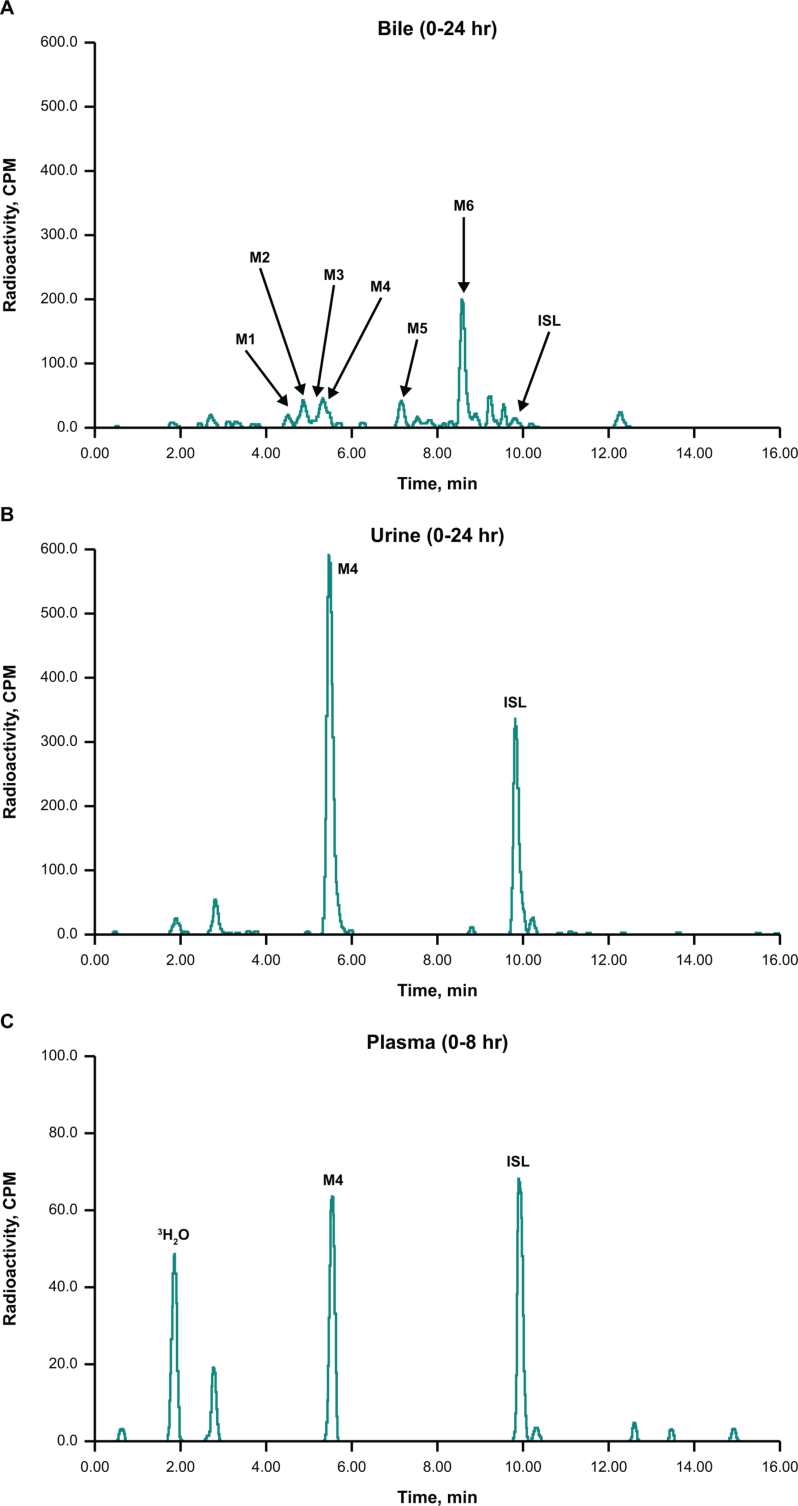
Representative radiochromatograms of pooled (**A**) bile, (**B**) urine, and (**C**) plasma from Wistar Hannover rats dosed orally with 5 mg/kg [^3^H]ISL. CPM, counts per minute; LC-HRMS, liquid chromatography HRMS. All metabolites, except those that could not be detected by LC-HRMS due to their small quantities or low ionization potential, were identified based on radiochromatographic profile, exact mass, and fragmentation pattern.

Metabolism via oxidative deamination was the primary mechanism of elimination in rats, in which M4 was the major metabolite in excreta and accounted for 43.1% of the administered dose (Table 2). The radioactivity recovered in bile and urine was mostly in the form of metabolites (Fig. 2A and B); however, in urine, a substantial portion of radioactivity was recovered as unchanged parent compound, accounting for 22.3% of the administered dose (Table 2). Glucuronide conjugates of M4 (M1, M2, M3, and M5) were minor metabolites in bile, each accounting for <1% of the administered dose (Table 2). A minor glucuronide of ISL (M6) was also detected in bile (Fig. 2A). In rat plasma, ISL, M1, and M4 were the only drug-related species identified by radiochemical and/or high-resolution mass spectrometry (HRMS) analysis in a pooled 0–8-hour plasma sample (Fig. 2C; Table 2).

**TABLE 2:** Summary of metabolites recovered in pooled plasma, urine, feces, and bile following single 5-mg/kg oral-dose administration of [^3^H]ISL in rats and monkeys^*a*^

Component	Percentage of total radioactivity (dose percentage)			
	Plasma (0–8 hours)	Urine (0–24 hours)	Feces (0–24 hours)	Bile (0–24 hours)
Wistar Hannover rats (*n* = 3)				
ISL	NQ^*b*^	22.3	NA	0.2
M1	NQ^*c*^	ND	NA	0.3
M2	ND	ND	NA	0.7
M3	ND	ND	NA	0.9
M4	NQ^*b*^	43.1	NA	0.1
M5	ND	ND	NA	0.5
M6	ND	ND	NA	2.6
Rhesus monkeys (*n* = 3)				
ISL	NQ^*b*^	34.3	2.7	NA
M4	NQ^*b*^	5.2	28.3	NA
M6	ND	4.8	ND	NA
M7	ND	0.9	ND	NA

^*a*^ HRMS, high-resolution mass spectrometry; LC-MS/MS, liquid chromatography with tandem mass spectrometry; NA, not analyzed; ND, not detected; NQ, not quantified.

^*b*^ Metabolites were detected by either radiometric or LC-MS/MS analysis; levels were not quantified.

^*c*^ Trace levels detected by HRMS only; levels were not quantified.

**Fig 3:**
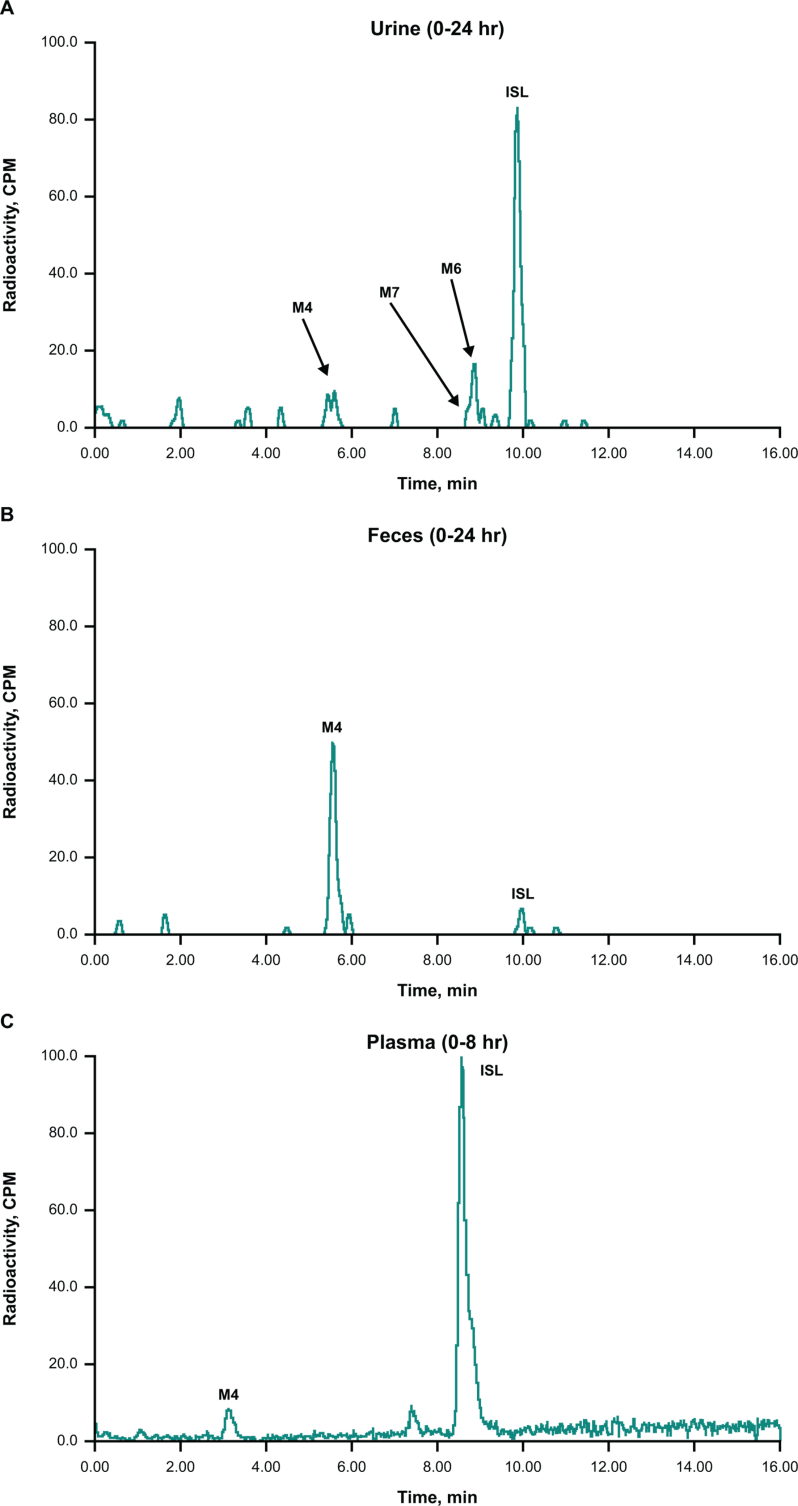
Representative radiochromatograms of pooled (**A**) urine and (**B**) feces and LC-HRMS extracted ion chromatogram of (**C**) pooled plasma from rhesus monkeys dosed orally with 5 mg/kg [^3^H]ISL. LC-HRMS, liquid chromatography HRMS. All metabolites, except those that could not be detected by LC-HRMS due to their small quantities or low ionization potential, were identified based on radiochromatographic profile, exact mass, and fragmentation pattern.

In rhesus monkeys, ISL was the major species in urine and represented 34.3% of the administered dose. M4 and glucuronide conjugates of ISL, M6 and M7, were minor metabolites in urine (Fig. 3A; Table 2). In feces, M4 was the major metabolite (Fig. 3B), accounting for 28.3% of the administered radioactivity, and ISL was a minor component (Table 2). Bile was not profiled due to the relatively low recovery (3.6% of the administered radioactivity; Table 1) in this matrix. In monkey plasma, no discernable peaks were identified in the radiochemical analysis of a pooled 0–8-hour plasma sample; however, both ISL and M4 were identified by HRMS analysis in the same sample (Fig. 3C).

### Plasma and intracellular PK in nonclinical species

**Fig 4:**
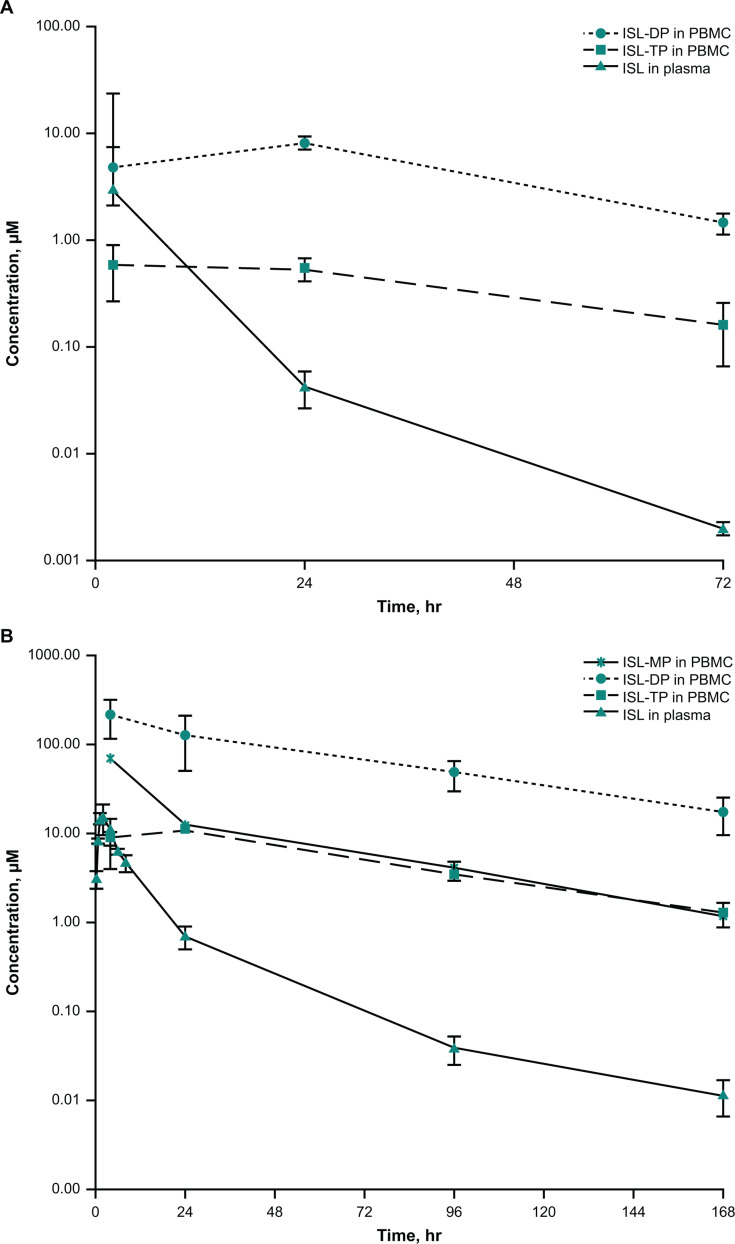
Concentration-time profile of ISL in plasma and ISL-­MP, -­DP, and -­TP in PBMCs following oral administration of ISL in (**A**) rats and (**B**) monkeys. (Mean ± SD; *n* = 3). ISL-MP levels were below the lower limit of quantitation at all time points.

The concentration-time profiles and mean PK parameter values for ISL in plasma and for ISL-mono-, di-, and triphosphates (ISL-MP, -DP, and -TP) in peripheral blood mononuclear cells (PBMCs) following oral administration to rats are shown in Fig. 4A and summarized in Table 3, respectively. Levels of ISL in plasma were measurable for ≤72 hours but were below the lower limit of quantification (LLOQ) by the 168-hour time point. ISL plasma concentrations were maximal at 2 hours postdose and declined in a biphasic manner until the 72-hour time point, with an approximate terminal half-life (*t*_½_) of 10 hours (Table 3). The intracellular concentration of ISL in rat PBMCs was measurable only at 2 hours postdose. Levels of ISL-MP in PBMCs were below the LLOQ for all time points. ISL-DP and ISL-TP in PBMCs were measurable for ≤72 hours but below the LLOQ at the 168-hour time point (Fig. 4A). The apparent intracellular levels of ISL-DP increased from 2 to 24 hours but declined through 72 hours, with a *t*_½_ of 20 hours. The apparent intracellular levels of ISL-TP were constant between 2 and 24 hours postdose but decreased over the 24–72-hour time frame, with a *t*_½_ of 25 hours (Fig. 4A; Table 3).

**TABLE 3:** Mean PK parameter values for ISL and ISL-MP, -DP, and -TP in plasma and PBMCs following oral administration of ISL in rats and monkeys^*a*^^,^^*f*^^,^^*g*^

Rat (30 mg/kg ISL; *n* = 3)^*b*^							
Matrix	Analyte	AUC_0–72_ (µM•h)	Dose-normalized AUC_0–72_	*C*_max_ (µM)	Dose-normalized C_max_	*T*_max_ (h)	*t*_½_ (h)
Plasma	ISL	20.0	0.67	3.0	0.10	2.0	10.0
PBMC^*c*^	ISL	NC^*d*^	NC	66.0	2.2	2.0	NC
	ISL-MP	NC^*d*^	NC	NC	NC	NC	NC
	ISL-DP	330.0	11.0	8.1	0.27	24.0	20.0
	ISL-TP	30.0	1.0	0.6	0.02	2.0	25.0

Monkey (50 mg/kg; *n* = 3) mean (SD)^*e*^							
Matrix	Analyte	AUC_0–168_ (µM•h)	Dose-normalized AUC_0–168_ (µM•h)	*C*_max_ (µM)	Dose-normalized *C*_max_ (µM)	*T*_max_ (h)	*t*_½_ (h)
Plasma	ISL	128.0 (7.0)	2.6	16.5 (5.2)	0.33	1.3 (0.6)	41.0 (6.0)
PBMC^*c*^	ISL	NC^*d*^	NC	5.9 (2.9)	0.12	4.0 (0.0)	NC
	ISL-MP	1,600.0 (900)	32	70.0 (27.0)	1.40	4.0 (0.0)	21.0 (15.0)
	ISL-DP	11,700.0 (3,600)	234	260.0 (30.0)	1.0	11.0 (12.0)	48.0 (4.0)
	ISL-TP	850.0 (40.0)	17	12.0 (1.0)	0.24	17.0 (12.0)	49.0 (8.0)

^*a*^ AUC_0–72_, area under the concentration-time curve from 0 to 72 hours; AUC_0–168_, area under the concentration-time curve from 0 to 168 hours; *C*_max_, maximum concentration; NC, not calculated; *t*_½_, terminal half-life; *T*_max_, time to maximum concentration.

^*b*^ Data represent mean values of *n* = 3 from terminal sampling; apparent AUC and *t*_½_ values were reported based upon sparse sampling.

^*c*^ Due to the insufficient stabilization of the PBMC samples, it is highly likely that ISL-TP hydrolyzed to the more stable ISL-DP during sample storage and preparation for bioanalysis. Thus, levels of ISL-DP are likely overestimated, and levels of ISL-TP are likely underestimated.

^*d*^ Not calculated due to insufficient data.

^*e*^ Data represent mean values of *n* = 3 ± SD; estimated AUC and *t*_½_ values were reported based upon sparse sampling of PBMCs.

^*f*^ Concentrations of ISL may be converted to nanogram per milliliter by multiplying values expressed in micromolar by 293.3.

^*g*^ Concentrations in picomole per million PBMCs were converted to micromolar using a cell volume of 0.200 pL/PBMC.

The concentration-time profiles and mean PK parameter values for ISL in plasma and ISL-MP, -DP, and -TP in PBMCs following oral administration to monkeys are shown in Fig. 4B and summarized in Table 3. ISL exhibited rapid absorption: time to maximum concentration (*T*_max_) was 1.3 hours postdose; concentrations declined in a multiphasic manner, with a *t*_½_ of 41 hours, approaching the intracellular half-lives of the phosphorylated anabolites (Fig. 4B; Table 3). The intracellular concentration of ISL in PBMCs was only measurable at 4 hours postdose. The apparent intracellular levels of ISL-DP were significantly higher than those of ISL-MP and -TP (Table 3).

### *In vitro* metabolism

**Fig 5:**
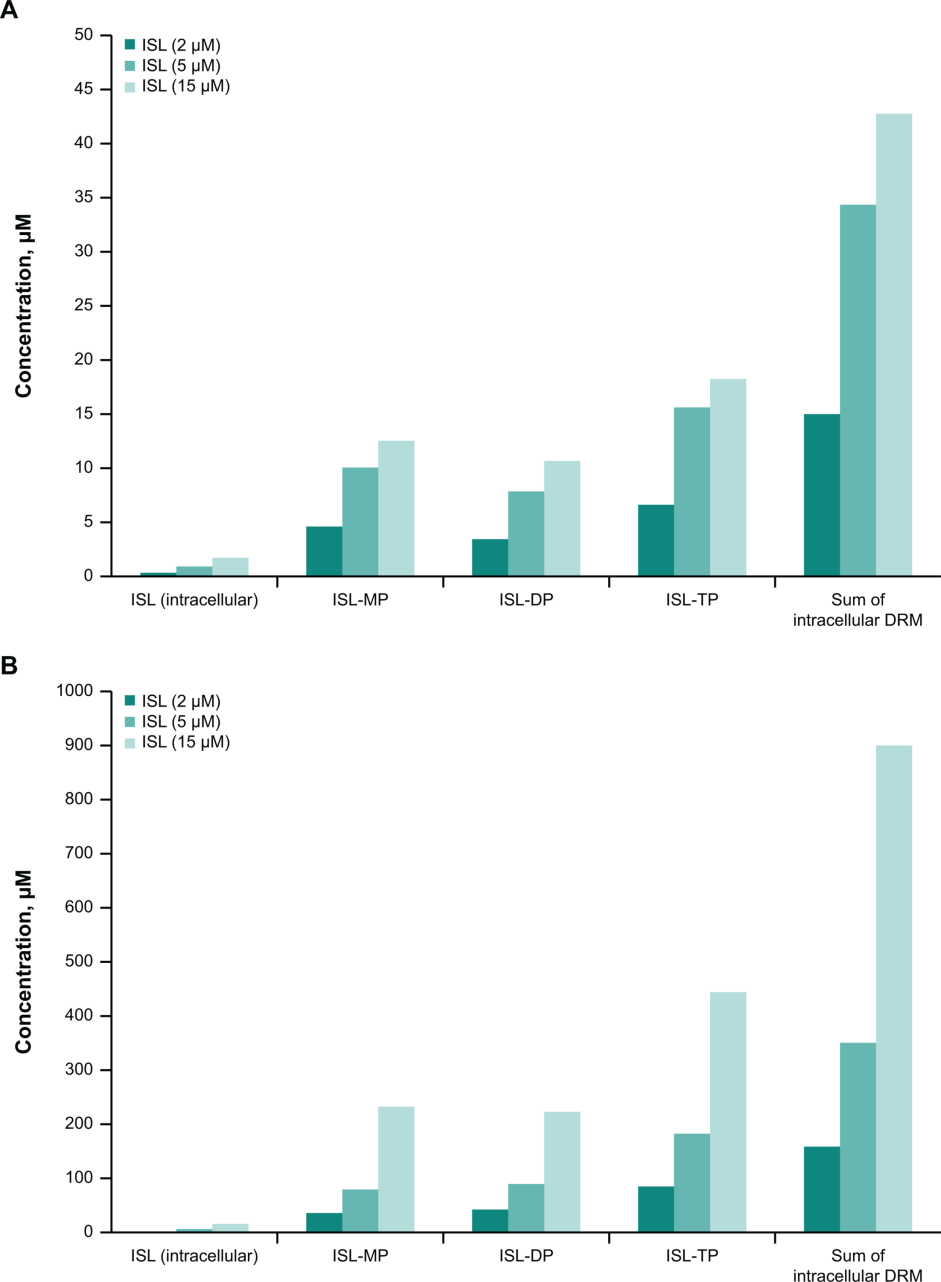
Intracellular concentrations of ISL, ISL-MP, -DP, and -TP anabolites in PBMCs following incubation of ISL (2, 5, and 15 µM) in fresh (**A**) monkey and (**B**) human blood for 2 hours. DRM, drug-related material.

In *ex vivo* blood incubation assays, the incubation time of 2 hours was deemed sufficient to establish equilibrium based on equivalent concentrations of intracellular ISL concentrations in PBMCs and extracellular ISL. In both monkey and human PBMCs, intracellular levels of total ISL drug-related material increased linearly with increasing extracellular ISL concentrations (Fig. 5A and B). Phosphorylation of ISL was efficient, and intracellular levels were in the order of ISL-TP > ISL-MP ~ ISL-DP >> ISL. The ratio of ISL-TP to ISL-DP was approximately 2:1. The uptake of ISL into human PBMCs was more efficient than in monkeys: levels of total ISL drug-related material after a 2-hour incubation were consistently 10-fold higher in human PBMCs than in monkey PBMCs, irrespective of the initial ISL concentrations (Fig. 5A and B).

### Clinical study demographics and safety

A total of six male participants in general good health without HIV infection were enrolled, and all six completed the study. The mean age was 38 years (range, 29–54 years) with a mean body mass index of 26.7 kg/m^2^ (range, 20.5–30.9 kg/m^2^). Four participants identified as Black, one as Asian, and one as White; none identified as Hispanic or Latino. No adverse events or discontinuations were reported. No findings of significance with respect to vital signs, electrocardiograms, or laboratory safety results were observed.

### Excretion of radioactivity and metabolite profile in humans

**Fig 6:**
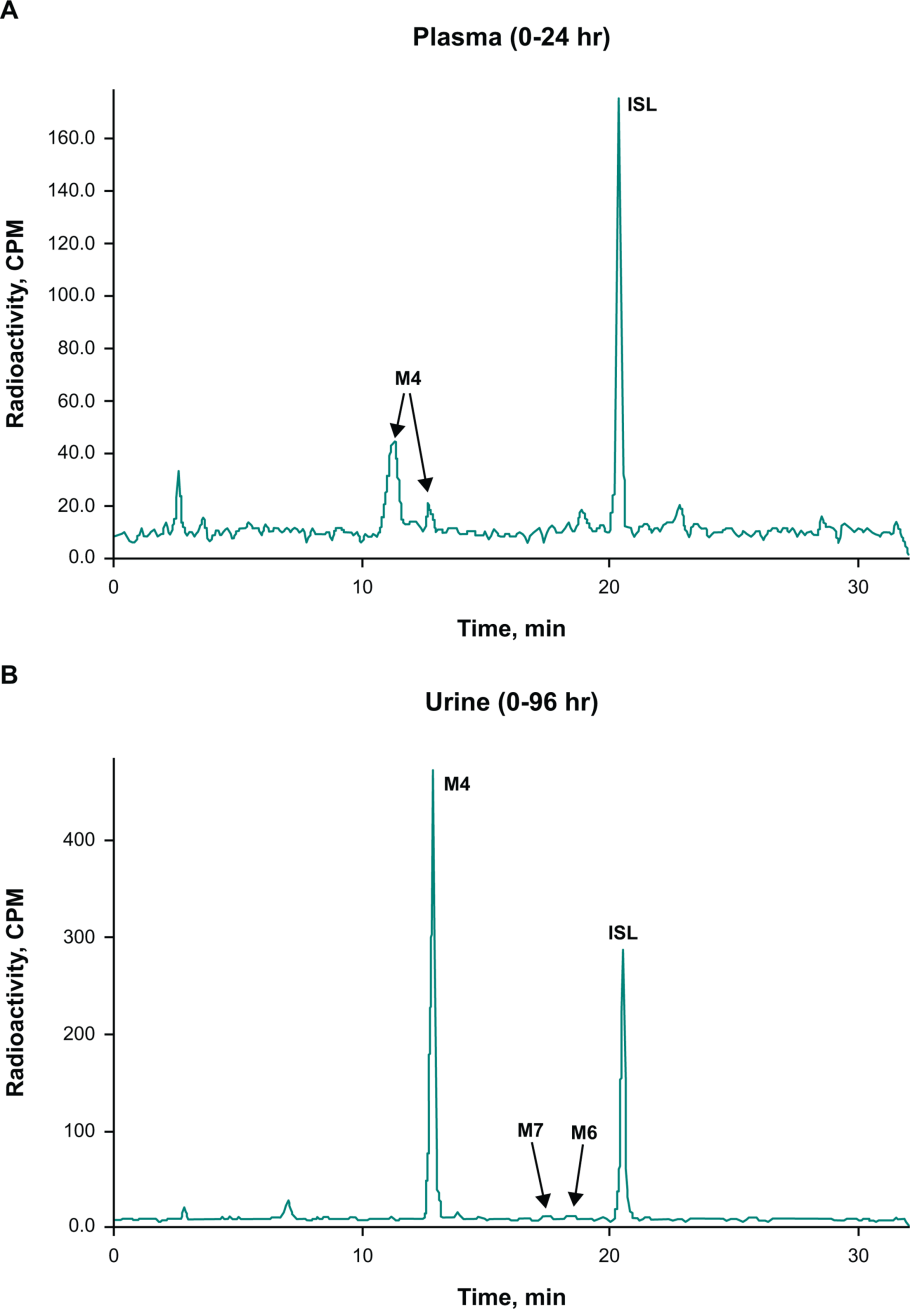
Representative radiochromatograms of pooled (**A**) plasma and (**B**) urine following single-dose administration of 10 mg [^14^C]ISL in healthy male participants. AUC_0–24_, area under the concentration-time curve from 0 to 24 hours; CPM, counts per minute. Pooled plasma (0–24 hours in proportion to AUC_0–24_). In plasma, the main ISL metabolite, M4, was observed as two peaks; the splitting was attributed to a matrix effect. In urine, ISL metabolites M6 and M7 were detected by HRMS analysis only.

Following oral administration of [^14^C]ISL in humans, 97.7% of the radioactive dose was recovered within 336 hours, with 91.4% recovered in urine and 6.3% recovered in feces (Table 4). In human plasma, ISL was the major circulating species (58% of the total radioactivity in plasma), with M4 as the major metabolite (31%; Table 5; Fig. 6A). In urine, most of the radioactivity was assigned to M4 (Fig. 6B), accounting for 53% of the administered dose (Table 5), while unchanged ISL accounted for 32% of the dose (Fig. 6B; Table 5). Glucuronide conjugates of ISL, M6 and M7, were observed in trace quantities in urine.

**TABLE 4:** Arithmetic mean percentage of radioactive dose recovered following single-dose administration of 10 mg [^14^C]ISL in healthy male participants (*N* = 6)^*a*^

	Urine	Feces	Total
Fraction of radioactivity recovery, mean % (95% CI)	91.4 (87.9–95.0)	6.3 (3.7–8.9)	97.7 (96.4–99.0)

^*a*^ CI, confidence interval.

**TABLE 5:** Summary of metabolites recovered in pooled plasma, urine, and feces following single-dose administration of 10 mg [^14^C]ISL in healthy male participants^*a*^

Component	Percentage of total radioactivity (dose percentage)		
	Plasma (0–24 hours)	Urine (0–96 hours)^*b*^	Feces (0–144 hours)
ISL	58	35 (32.0)	ND
M4	31	58 (53.0)	NQ^*c*^
M6	ND	NQ^*c*^	ND
M7	ND	NQ^*c*^	ND
Unidentified metabolites	<7	1 (0.9)	ND

^*a*^ ND, not detected; NQ, not quantified.

^*b*^ Values in parentheses represent the percentage of the dose calculated using the total percentage of dose recovered in urine over 0–336 hours.

^*c*^ Metabolites were detected by HRMS only; levels were not quantified.

### Plasma and intracellular PK in humans

In humans, following a single oral dose of ISL, median plasma *T*_max_ was 0.50 hours for both ISL and total radioactivity (Table 6). Due to limitations of the bioanalytical assay, the ISL and radioactivity terminal elimination phases could not be characterized; therefore, area under the plasma concentration-time curve from 0 to infinity (AUC_0–∞_) and *t*_½_ are not reported.

**TABLE 6:** Summary statistics of plasma ISL and radioactivity following single-dose administration of 10 mg [^14^C]ISL in healthy male participants (*N* = 6)^*a*^^,^^*d*^^,^^*e*^

Parameter	GM (95% CI)
ISL AUC_0–24_^*b*^ (μM•h)	0.675 (0.617–0.740)
Total radioactivity AUC_0–24_^*b*^ (μMEq•h)	2.29 (2.09–2.51)
ISL *C*_max_^*b*^ (µM)	0.263 (0.217–0.319)
Total radioactivity *C*_max_^*b*^ (µMEq)	0.739 (0.610–0.898)
ISL *C*_24_^*b*^ (µM)	0.00368 (0.00331–0.00413)
Total radioactivity *C*_24_^*b*^ (µMEq)	0.0339 (0.0305–0.0380)
ISL *T*_max_^*c*^ (h)	0.50 (0.50, 0.50)
Total radioactivity *T*_max_^*c*^ (h)	0.50 (0.50, 0.50)

^*a*^ AUC_0–24_, area under the concentration-time curve from 0 to 24 hours; *C*_24_, concentration at 24 hours; CI, confidence interval; *C*_max_, maximum concentration; GM, geometric least-squares mean; NA, not applicable; *T*_max_, time to maximum concentration.

^*b*^ Back-transformed least-squares mean and CI from linear mixed-effects model were performed on natural log-transformed values.

^*c*^ Median (minimum, maximum) is reported.

^*d*^ Concentrations of ISL may be converted to nanogram per milliliter by multiplying values expressed in micromolar by 293.3.

^*e*^ Concentrations of total radioactivity may be converted to nanogram equivalent per milliliter by multiplying values expressed in micromolar equivalent by 295.

## DISCUSSION

ISL is an investigational deoxyadenosine analog with promising clinical efficacy data that shows potential in the treatment of HIV-1 (3, 4, 6, 10). Due to the importance of fully understanding the metabolic profile and disposition of new agents, here, we have characterized the absorption, metabolism, and excretion of ISL in nonclinical studies and a clinical study.

ISL was readily absorbed in all species following oral administration, as demonstrated by the radioactivity recovered in urine in humans and in urine and bile in rats and monkeys. Consistent with the exclusive recovery of ISL-related radioactivity in the urine in humans, the highest recovery of radioactivity in urine was seen in humans. The urine recovery was lower in rats, followed by monkeys. Parent ISL and metabolite M4 were the primary circulating ISL drug-related components and were prominently seen in urine and feces in all species.

In human plasma, ISL accounted for 58% of the circulating radioactivity, with M4 accounting for 31% of the radioactivity in plasma. In human urine, M4 accounted for the majority of the administered dose (53%), followed by ISL (32%). With respect to the administered dose, the relative amounts of ISL and M4 recovered in excreta varied across species. Fecal elimination of drug components was more prominent in monkeys vs rats or humans, although urinary elimination of the radioactive dose over fecal elimination was still dominant in monkeys. The proportion of M4 in monkey urine was also less than that in humans and rats, with M4 being the primary drug-related component in monkey feces. The presence of M4 in monkey feces indicated good absorption and suggested significant gut metabolism in this species, in which M4 is formed by oxidative deamination by adenosine deaminase (12), an enzyme with high expression in the gut (16, 17). The presence of ISL and M4 in excreta from all evaluated species indicated that ISL was eliminated primarily by a combination of renal excretion of unchanged drug and oxidative deamination to metabolite M4. In addition to M4, other ISL metabolites were observed, which include glucuronide conjugates of M4, as well as minor ISL glucuronide conjugates (M6 and M7; Fig. 1). However, most minor metabolites were present only in trace amounts (Fig. 1).

Antiviral activity of ISL is achieved through uptake into infected cells with subsequent phosphorylation to ISL-TP. Cellular uptake is assessed through measurement of the phosphorylated species in PBMCs. The PK of the ISL phosphorylated forms (ISL-MP, -DP, and -TP) was directly assessed in rats and monkeys; assessment of PBMCs in the clinical study was not feasible due to the challenges of isolating radiolabeled PBMCs. Efficient intracellular conversion of the parent drug was readily observed; however, quantitative assessment is limited owing to insufficient stabilization of the PBMC samples for both the rat and monkey studies. It was observed during method development (data not shown) that, without sample stabilization, ISL-TP hydrolyzes to the more stable ISL-DP form during sample storage. Therefore, it is likely that for these initial rat and monkey studies, ISL-TP hydrolyzed during sample storage resulting in an underestimation of ISL-TP concentrations and an overestimation of ISL-DP levels. Uptake and conversion were additionally assessed *in vitro* via direct incubation of ISL in PBMCs (monkey and human). ISL cellular uptake was seen, with efficient phosphorylation to the mono-, di-, and triphosphate forms. The active ISL-TP form was most prominent. The extent of ISL uptake into human PBMCs was more efficient than in monkey PBMCs; levels of total ISL drug-related material were consistently 10-fold higher in human PBMCs than in monkey PBMCs, irrespective of the initial ISL concentration. Comparison of the monkey and human results suggests a potential species difference in intracellular ISL uptake and is consistent with previous reports (18). This species difference may pose challenges when using monkeys as nonclinical species to predict ISL and ISL-TP concentrations in humans.

These studies characterized the absorption, metabolism, and elimination of ISL; however, limitations are acknowledged. Sample stability issues limited the profiling of the ISL-MP, -DP, and -TP intracellular PK in rats and monkeys. Assay sensitivity yielded issues with monkey plasma radioactivity assessments and with the human ISL PK and radioactivity assessments. It is also acknowledged that the nonclinical and clinical studies had small sample sizes with only single-dose administration of ISL. Based on multiple-dose ISL PK data, single-dose administration should be predictive of multiple-dose behavior (3). In the human study, the geometric mean ratio of ISL to total radioactivity could not be effectively evaluated because data older than 24 hours were not evaluable due to the limitation of the bioanalytical assay. These data would have provided further evidence regarding the degree of circulating parent ISL.

Across nonclinical species and humans, ISL is well absorbed following oral administration. ISL and the metabolite M4 are the major circulating drug components across species, and ISL and M4 are the primary drug-related components in excreta. ISL is readily taken up cellularly, with efficient phosphorylation to the mono-, di-, and triphosphate forms. The pharmacologically active ISL-TP is the primary intracellular phosphorylated species. Clinical development of oral once-monthly ISL for HIV pre-exposure prophylaxis has been discontinued due to dose-dependent decreases in total lymphocyte and CD4+ T-cell counts seen across the ISL development program (19, 20). However, ISL continues to be investigated as part of a two-drug HIV-1 treatment regimen with doravirine (at a dose of 0.25 mg once daily; NCT05631093) and with lenacapavir (at a dose of 2 mg once weekly; NCT05052996) (20, 21). These lower doses of ISL have not been associated with decreases in total lymphocyte or CD4+ T-cell counts. This characterization of the absorption, metabolism, and elimination of ISL supports the further development of ISL for the treatment of HIV-1.

## MATERIALS AND METHODS

### Chemicals

For the nonclinical rat and monkey excretion and mass balance study, [^3^H]ISL was synthesized by Process Chemistry, Merck & Co., Inc., Rahway, NJ, USA, with a specific activity of 2.464 µCi^−1^ (0.7228 Ci^−1^) and radiochemical purity of 97%. For the rat excretion and mass balance study, unlabeled ISL was combined with [^3^H]ISL in a solution of [^3^H]ISL with a nominal concentration of 1.0 mg/mL and a measured specific activity of approximately 72.5 µCi/g. For monkeys, the oral dosing solution (1.0 mg/mL) had a nominal specific activity of approximately 1.4 µCi/g. For the human excretion and mass balance study, [^14^C]ISL was synthesized by Analytical Research and Development, Merck & Co., Inc., Rahway, NJ, USA and was supplied as an oral suspension (10 mg/mL) with a specific activity of approximately 6.2 µCi/mg (0.002 µCi^−1^) and radiochemical purity of 100%.

### *In vivo* animal studies

All animal studies were conducted using protocols in accordance with the Institutional Animal Care and Use Committee at Merck & Co., Inc., Rahway, NJ, USA, which adhere to the regulations outlined in the US Department of Agriculture Animal Welfare Act.

Nonclinical assessment of ISL radioactivity mass balance, metabolic profiling, and PK assessments was performed on rats and monkeys. Bile duct-cannulated male Wistar Hannover rats (*n* = 3) weighing 300–360 g and intact male Wistar Hannover rats (*n* = 3) weighing 280–300 g with cannulated jugular veins were fasted overnight then dosed with 5 mL/kg [^3^H]ISL (5 mg/kg; approximately 100 µCi per animal). Bile, urine, and fecal samples were collected from the bile duct-cannulated animals, and blood was collected from the intact animals through 72 hours postdose; cages were rinsed and wiped for radioactivity analysis at the end of the study. Bile duct-cannulated male rhesus monkeys (*n* = 3) weighing 6.9–9.2 kg were fasted overnight then dosed via oral gavage with 5 mL/kg [^3^H]ISL (5 mg/kg; approximately 60 µCi per animal). Bile was collected through 72 hours postdose; blood samples were collected through 96 hours postdose; and urine and fecal samples were collected through 120 hours postdose. Cages were rinsed at intervals up to 120 hours postdose.

### *In vivo* metabolism studies

For PBMC distribution and anabolism assessment in rats and monkeys, male Wistar Hannover rats (*n* = 12) weighing approximately 300 g and male rhesus monkeys (*n* = 3) weighing 4.5–5.0 kg were given an oral suspension of 5 mL/kg ISL at final doses of 30 mg/kg and 50 mg/kg, respectively. Blood samples were collected for plasma and PBMC analysis through 168 hours postdose.

### *In vitro* metabolism studies

Fresh heparinized human and monkey blood samples were collected from at least three donors (each species). Blood was incubated with ISL (2, 5, and 15 µM) at 37°C for 2 hours. After incubation, PBMCs were isolated and analyzed for intracellular levels of ISL, ISL-MP, -DP, and -TP.

### Clinical study

Study participants (*n* = 6) were healthy adult males aged 18–55 years with a body mass index ≥18 and ≤32 kg/m^2^. All participants signed written informed consent before study entry. Participants were enrolled between 26 February 2020 and 26 May 2021. Participants received a single oral dose of 10 mg (approximately 62 µCi) [^14^C]ISL in the fasted state. Whole blood, plasma, urine, fecal samples, and toilet tissue were collected throughout the study for radioactivity mass balance, metabolic profiling, and PK profiling. Blood was collected through 120 hours postdose; urine and fecal samples were collected through 336 hours postdose. Clinical safety was monitored.

### Measurement of radioactivity

All samples collected in the animal and human metabolic profiling studies were analyzed for total radioactivity content by liquid scintillation counting. Actual doses administered in disintegrations per minute were used to calculate the percentage of dose recovered.

### LC-HRMS and radiochromatographic analysis

Aliquots of urine were pooled proportionally relative to the total volume collected over 24 hours for rats and monkeys and pooled over 96 hours for humans. After centrifugation, the resulting supernatant was analyzed by liquid chromatography (LC)-HRMS with radiometric detection.

For rats and monkeys, bile samples from the 0- to 24-hour collection interval were pooled, mixed with acetonitrile, vortexed, and sonicated. After centrifugation, the resulting supernatants were analyzed by radiometric LC-HRMS. Fecal homogenate samples from 0 to 24 hours were pooled. Aliquots of fecal homogenate were mixed with methanol, vortexed, and centrifuged, and the resulting supernatant was analyzed by radiometric LC-HRMS. Recovery of radioactivity in the reconstituted samples was measured using liquid scintillation counting.

For humans, aliquots of fecal samples were pooled proportionally relative to weight across individuals to generate a single fecal homogenate. The sample was mixed with acetonitrile, vortexed, and centrifuged, and the resulting supernatant was analyzed by LC-HRMS with offline radiometric profiling.

Plasma samples from 0 to 8 hours in rats and monkeys and 0 to 24 hours for humans were pooled for each species according to the Hamilton time proportional pooling algorithm (22) to yield one sample that had a concentration proportional to the PK AUC. An aliquot of the pooled sample was treated by protein precipitation with the addition of acetonitrile or acetonitrile plus methanol [90/10 (vol/vol)] followed by vortex mixing and centrifugation. The resulting supernatants were analyzed by radiometric LC-HRMS. The LLOQ for radiometric detection was approximately 10–20 counts per minute.

### Analysis of islatravir in plasma

Following extraction with acetonitrile, the concentrations of ISL in rat and monkey plasma were determined by liquid chromatography with tandem mass spectrometry (LC-MS/MS) analysis with a dynamic range of 5–5,000 nM. Human plasma was extracted with methyl-*tert*-butyl ether, and ISL levels were determined using a validated LC-MS/MS method. The analytical range of this method was 0.0682–68.2 nM (20.0–20000.0 pg/mL).

### Analysis of the intracellular levels of ISL and ISL-MP, -DP, and -TP in PBMCs

PBMCs were isolated from heparinized rat, monkey, and human blood samples by density gradient centrifugation. PBMCs collected from *in vitro* studies were stabilized with 70% methanol before freezing, whereas PBMCs collected from monkeys and rats in *in vivo* studies were not. The intracellular concentrations of ISL, ISL-MP, -DP, and -TP were determined by an LC-MS/MS assay, for which separation of the ISL phosphate anabolites was achieved by ion exchange chromatography. The LLOQ was between 2 and 13 nM depending on the volumes of cell suspension used in the assay. Analyte concentrations in cell samples across species were determined by weighted linear regression of the standard curve and normalized to the total viable PBMC cell counts. Concentrations in units of picomole per million PBMCs were converted to micromolar using a cell volume of 0.200 pL per PBMC (23, 24).

### PK analysis

PK parameter values were calculated using noncompartmental methods in Watson LIMS (Thermo Fisher Scientific; Waltham, MA, USA) for the nonclinical studies and Phoenix WinNonlin version 8.1 (Certara; Princeton, NJ, USA) for the clinical study. The AUCs from 0 to a specific time, such as 24 hours, 72 hours, and so on (AUC_0–24_ and AUC_0–72_), were calculated using the linear trapezoidal method for ascending concentrations and the log trapezoidal method for descending concentrations. The maximum plasma concentration, concentration at 24 hours, and *T*_max_ were obtained by graphical inspection of the plasma concentration-time data. The *t*_½_ in nonclinical species was determined by selected time points based on visual inspection of the data.

### Statistical analysis

For the clinical study, prior to analysis, individual values of each PK parameter and analyte were natural log-transformed and evaluated by use of a linear mixed-effects model with analyte (ISL and total radioactivity) as a fixed effect and participant as a random effect. Model-based summary statistics, geometric means, and 95% confidence intervals were calculated. Arithmetic means (on the raw scale) and corresponding 95% confidence intervals for recovery of total radioactivity (as a percentage of dose administered) were provided for urine and feces, as well as in total.

## Data Availability

The data sharing policy, including restrictions, of Merck Sharp & Dohme LLC, a subsidiary of Merck & Co., Inc., Rahway, NJ, USA (MSD), is available at https://trialstransparency.merckclinicaltrials.com/policies-perspectives.aspx. Requests for access to the clinical study data can be submitted via email to dataaccess@msd.com.
